# JP4-039, a Mitochondria-Targeted Nitroxide, Mitigates the Effect of Apoptosis and Inflammatory Cell Migration in the Irradiated Mouse Retina

**DOI:** 10.3390/ijms25126515

**Published:** 2024-06-13

**Authors:** Jennifer O. Adeghate, Michael W. Epperly, Katherine Anne Davoli, Kira L. Lathrop, Peter Wipf, Wen Hou, Renee Fisher, Stephanie Thermozier, M. Saiful Huq, Jose-Alain Sahel, Joel S. Greenberger, Andrew W. Eller

**Affiliations:** 1 Department of Ophthalmology, University of Pittsburgh School of Medicine and UPMC Vision Institute, UPMC Mercy Pavilion, Pittsburgh, PA 15219, USA; 2 Department of Radiation Oncology, UPMC Hillman Cancer Center, Pittsburgh, PA 15232, USA; 3 Department of Bioengineering, Swanson School of Engineering, University of Pittsburgh, Pittsburgh, PA 15260, USA; 4 Department of Chemistry, University of Pittsburgh, Pittsburgh, PA 15260, USA; 5 Department of Pharmaceutical Sciences, School of Pharmacy, University of Pittsburgh, Pittsburgh, PA 15261, USA; 6 Department of Ophthalmology and Visual Sciences, University of Illinois at Chicago, Chicago, IL 60612, USA

**Keywords:** radiation, retinopathy, toxicity, apoptosis, antioxidant, intravitreal, injection, inflammatory

## Abstract

We hypothesize that the injection of JP4-039, a mitochondria-targeted nitroxide, prior to irradiation of the mouse retina may decrease apoptosis and reduce neutrophil and macrophage migration into the retina. In our study, we aimed to examine the effects of JP4-039 in the mouse retina using fluorescent microscopy, a terminal deoxynucleotidyl transferase dUTP nick end labeling (TUNEL) assay, and flow cytometry. Forty-five mice and one eye per mouse were used. In Group 1, fluorescent microscopy was used to determine retinal uptake of 10 µL (0.004 mg/µL) of intravitreally injected BODIPY-labeled JP4-039 at 0, 15, and 60 min after injection. In Group 2, the TUNEL assay was performed to investigate the rate of apoptosis after irradiation in addition to JP4-039 injection, compared to controls. In Group 3, flow cytometry was used to determine the extent of inflammatory cell migration into the retina after irradiation in addition to JP4-039 injection, compared to controls. Maximal retinal uptake of JP4-039 was 15 min after intravitreal injection (*p* < 0.0001). JP4-039-treated eyes had lower levels of retinal apoptosis (35.8 ± 2.5%) than irradiated controls (49.0 ± 2.7%; *p* = 0.0066) and demonstrated reduced migration of N1 cells (30.7 ± 11.7% vs. 77.7 ± 5.3% controls; *p* = 0.004) and M1 cells (76.6 ± 4.2 vs. 88.1 ± 3.7% controls, *p* = 0.04). Pretreatment with intravitreally injected JP4-039 reduced apoptosis and inflammatory cell migration in the irradiated mouse retina, marking the first confirmed effect of this molecule in retinal tissue. Further studies may allow for safety profiling and potential use for patients with radiation retinopathy.

## 1. Introduction

Radiation retinopathy is a vision-threatening condition that can occur as a complication of external beam therapy and other forms of radiation in patients who have undergone treatment for ocular, orbital, and craniofacial malignancies [1,2,3]. Although radiation commonly affects structures such as the cornea, crystalline lens, and optic nerve, one of the most severe complications involves retinopathy and its subsequent therapeutic challenges [4]. Doses as low as 11 Gray (Gy; 1 Gy = 1 Joule/kilogram = 100 rad) have been found to cause radiation retinopathy [1,5], with the likelihood of damage increasing significantly after 25 Gy [6]. Radiation retinopathy is defined as acute if retinal changes occur within 6 h of irradiation, and chronic if clinical manifestations develop between 6 months and 3 years after irradiation [1,4]. In a study by Biancotto et al., proliferative radiation retinopathy was found to occur in 5.8% of patients within 5 years, and in 7% of patients within 10–15 years after plaque brachytherapy [7].

Although the cellular changes caused by radiation retinopathy are similar to those which occur in diabetic retinopathy, the entity that primarily differentiates radiation retinopathy from the latter is atrophy of the retinal pigment epithelium (RPE) [1]. Acute retinal changes involve necrosis of the photoreceptor cells [8], nuclear pyknosis, and outer retinal edema [9], while chronic changes include proliferative retinopathy secondary to retinal capillary endothelial cell injury, thrombosis, and subsequent retinal ischemia [1,2,10]. These processes have been linked to the generation of reactive oxygen species (ROS) directly and indirectly from the interaction of radiation, water, mitochondria, and the lipids in cell membranes [11]. In addition to mitochondrial damage, ionizing radiation has also been shown to induce abnormal gene (glutathione peroxidase) and protein (e.g., caspase 3) expression in the retina [12].

Mitochondria are particularly sensitive to external factors such as medications, radiation, and even contrast materials [13]. Radioactive molecules, similar to glycation end-products in diabetic retinopathy, interact with retinal cells to dissipate reactive electrons, thereby damaging biomolecules, proteins, and DNA [14]. These products react further with water to produce yet more ROS, causing a vicious cycle leading to cellular apoptosis. Because retinal photoreceptors have many mitochondria, the retina is predisposed to a greater risk of damage when there is increased ROS production [14], particularly since impaired mitophagy leads to further imbalance in the cells’ oxidative state [15]. Although some protection against proliferative retinopathy can be achieved with panretinal photocoagulation [16] or intravitreal anti-vascular endothelial growth factor (VEGF) injections [17], the prevention of radiation retinopathy has not been well studied.

**Figure 1:**
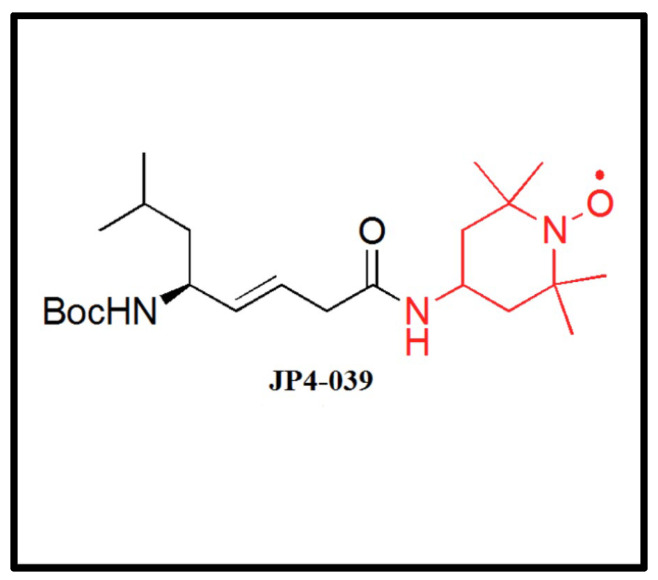
Molecular structure of JP4-039, a novel mitochondria-targeted nitroxide linked to an analog of gramicidin S.

JP4-039 is a novel, mitochondria-targeted nitroxide derived from the sequence of the cyclopeptide antibiotic gramicidin S (GS). This electron and ROS scavenger was first reported in 2009 by Rajagopalan et al. [18] (Figure 1), and was shown to mitigate radiation damage and apoptosis in irradiated cells [19]. We hypothesize that the injection of JP4-039 prior to the irradiation of mouse retina may decrease apoptosis and reduce neutrophil and macrophage migration into the retina. In our study, we aimed to examine these effects of JP4-039 on the mouse retina using fluorescent microscopy, a terminal deoxynucleotidyl transferase dUTP nick end labeling (TUNEL) assay, and flow cytometry.

## 2. Results

### 2.1. Retinal Uptake of JP4-039

**Figure 2:**
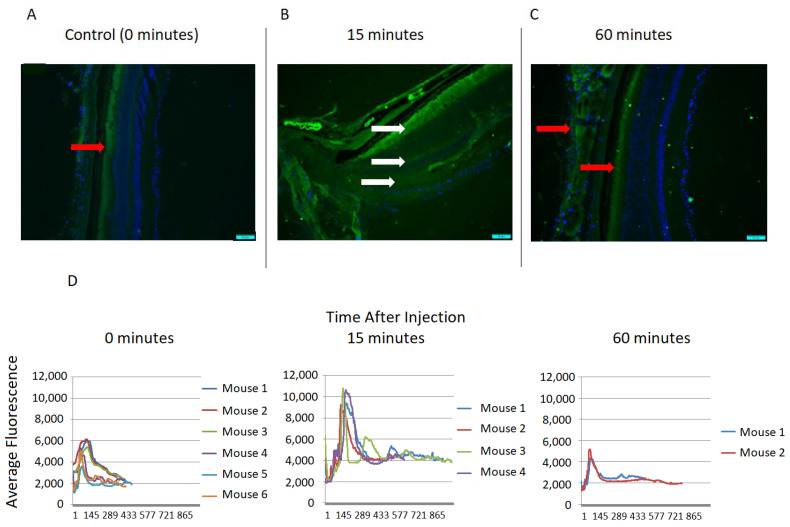

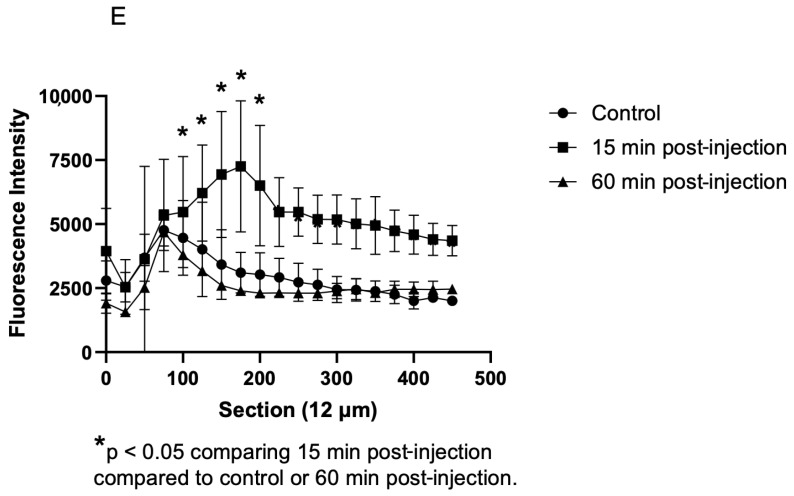
Retinal uptake of BODIPY-JP4-039. (**A**) Control retina with autofluorescence in the photoreceptors (red arrow). (**B**) Fluorescence in retina 15 min post-intravitreal injection of BODIPY-JP4-039: increased fluorescence in photoreceptor, outer nuclear, and inner nuclear layers and the presence of JP4-039 (white arrows), while the scleral autofluorescence remains. (**C**) Autofluorescence in the sclera and photoreceptors 60 min post-injection of BODIPY-JP4-039 (red arrows). Magnification: ×20. Scale bar = 50 µm. (**D**) Tracings of average fluorescence of all 12 µm retinal sections in the green channel are shown at 0 min, 15 min, and 60 min following injection of BODIPY-JP4-039. (**E**) There was a significant increase in retinal fluorescence at 15 min after JP4-039 injection (* *p* < 0.05), while there was no remaining fluorescence at 60 min. This demonstrates that there is an increase in the uptake of BODIPY-JP4-039 in the retina for at least 15 min following intravitreal injection.

After intravitreal injection of fluorescent BODIPY-labeled JP4-039 in Group 1, fluorescent microscopy showed the uptake of JP4-039 in the retina at 15 min post-injection, which dissipated by 60 min after injection (Figure 2A–C). The retina was then cut into serial sections of 12 µm spanning from the outer edge of the choroid to the inner retina, and the fluorescence of each section was measured. The fluorescence values for all the mice in each group were summarized as mean and standard error, and were plotted for each timepoint (Figure 2D). The fluorescence signal was significantly higher 15 min after BODIPY-labeled JP4-039 was injected intravitreally compared to 0 and 60 min post-injection (*p* < 0.05). This indicates that intravitreal administration results in the uptake of JP4-039 by the retinal cells for at least 15 min after injection. The level of JP4-039 was undetectable beyond 60 min after injection.

### 2.2. Apoptosis

**Figure 3:**
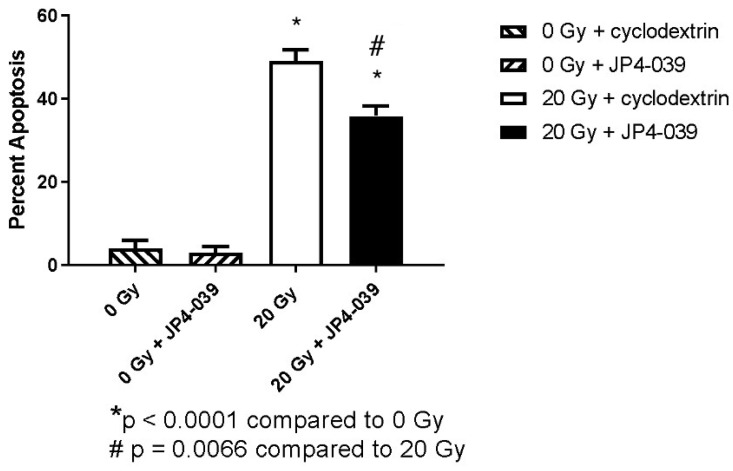
Injection of JP4-039 prevents apoptosis following irradiation. JP4-039 and cyclodextrin alone did not induce apoptosis in the absence of radiation (hashed bars). Irradiated mice injected with JP4-039 (solid black bar) had lower rates of apoptosis compared to vehicle-treated irradiated mice (solid white bar). Abbreviations: Gy = Gray. *: *p* < 0.0001 when comparing subgroups 2A (20 Gy + cyclodextrin) and 2B (20 Gy + JP4-039) to 2C (0 Gy+ JP4-039; control). #: *p* = 0.0066 when comparing subgroup 2B (20 Gy + JP4-039) to 2A (20 Gy + Cyclodextrin).

Neither the antioxidant JP4-039 nor the vehicle (cyclodextrin) in which it was dissolved induced apoptosis in the absence of irradiation (Figure 3, hashed bars). However, irradiation of eyes with 20 Gy caused a large and significant percentage of apoptotic cells in the mouse retina compared to mice that were not irradiated (*p* < 0.0001, Figure 3, solid white bar). Irradiated eyes that were injected intravitreally with JP4-039 had a significantly lower level of apoptosis in the retina compared to untreated eyes (35.8 ± 2.5% vs. 49.0 ± 2.7%, respectively, *p* = 0.0066, Figure 3, solid black bar).

### 2.3. JP4-039 and Migration of Proinflammatory Cells

**Figure 4:**
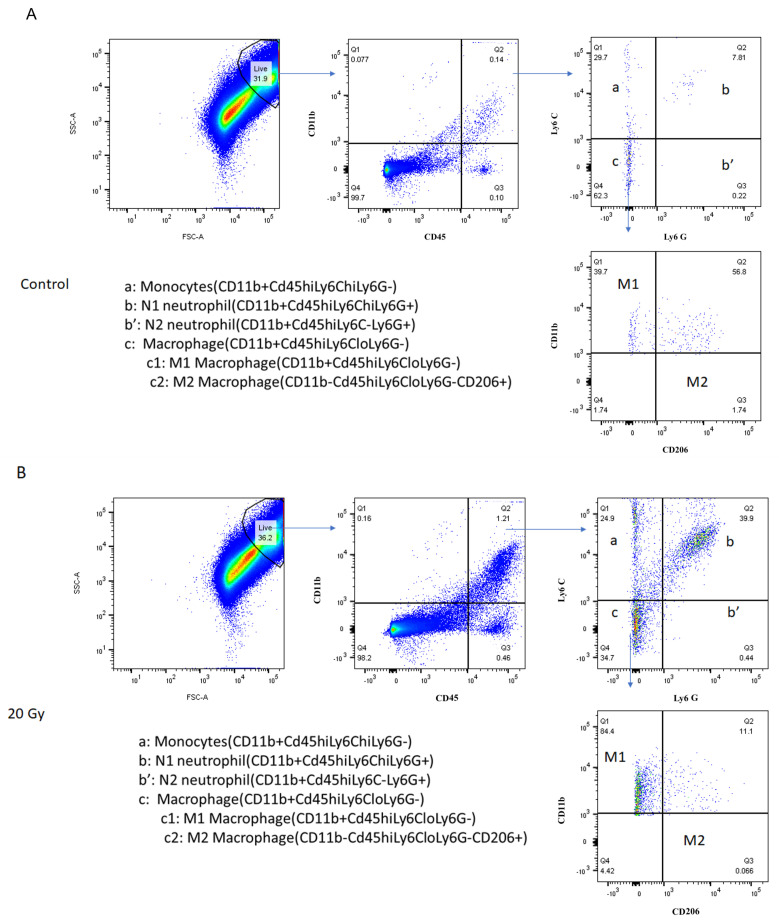

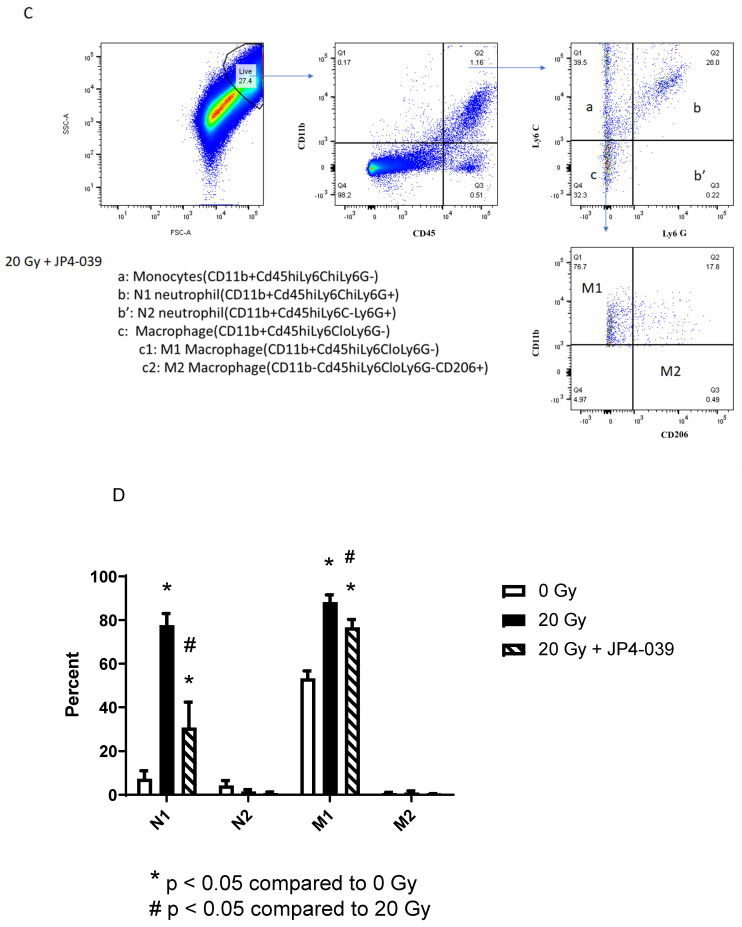
Intravitreal injection of JP4-039 decreases migration of neutrophils and macrophages. Flow cytometry analyses of retinal inflammatory cells obtained from (**A**) vehicle-only mice (31.9% live cells), (**B**) irradiated mice without treatment (36.2% live cells), and (**C**) irradiated mice treated with JP4-039 (27.4% live cells). (**D**) shows the results from the analysis of all 15 mice in Group 3. Mice injected with JP4-039 had a significantly lower percent of N1 (N1 hashed bar) and M1 (M1 hashed bar) cells compared to irradiation-only mice (solid black bars). Eyes with irradiation only had a significantly higher percentage of N1 (N1 solid black bar) and M1 (M1 solid black bar) cells when compared to unirradiated eyes. Abbreviations: Gy = Gray; SSC = Side Scatter; FSC = Forward Scatter. *: *p* < 0.05 when comparing irradiation-without-treatment (20 Gy + cyclodextrin) and irradiation-with-treatment (20 Gy + JP4-039) mice to treatment-only (0 Gy + JP4-039) mice. #: *p* < 0.05 when comparing irradiation-with-treatment mice (20 Gy + JP4-039) to irradiation-without-treatment mice (20 Gy + cyclodextrin).

Following irradiation, the retinas were removed from the irradiated eyes, made into a single cell suspension, and stained with antibodies against N1 and N2 neutrophils as well as M1 and M2 macrophages. The retinal cells were analyzed by flow cytometry as seen for control eyes (3A), irradiated and treated eyes (3B), and eyes injected with JP4-039 without irradiation (3C). Figure 4A–C are graphs from individual mice and are representative of the flow analysis of the retinal cell suspensions. The monocyte population was identified and the number and percentage of N1 and N2 neutrophils and M1 and M2 macrophages were determined in each subgroup. Irradiation of the eyes caused a large and significant increase in the percentage of neutrophils and macrophages by 120 h post-irradiation compared to unirradiated eyes (*p* = 0.05, Figure 4D, black bars). Table 1 shows the numbers of N1, N2, M1, and M2 cells for the percentages shown in Figure 4D.

Eyes treated with JP4-039 had a significantly lower amount of migration of N1 cells into the retina compared to those with irradiation alone (30.7 ± 11.7% vs. 77.7 ± 5.3%, respectively, *p* = 0.004, Table 1 and Figure 4D, N1 hashed bar). Similarly, JP4-039-injected eyes had a significantly lower migration of M1 cells into the retina compared to vehicle (76.6 ± 4.2% vs. 88.1 ± 3.7%, respectively, *p* = 0.0396, Table 1 and Figure 4D, M1 hashed bar). Eyes that received radiation only had a significantly higher percentage of N1 (77.7 ± 5.3% vs. 7.4 ± 3.6%, respectively, *p* = 0.0302, Figure 4D, N1 solid black bar) and M1 cells (88.1 ± 3.7% vs. 53.3 ± 3.4%, *p* = 0.0006, Figure 4D, M1 solid black bar) when compared to eyes that were not irradiated.

## 3. Discussion

JP4-039, a mitochondria-targeted GS-Nitroxide, is an antioxidant molecule that has previously been found to mitigate radiation damage in various organ systems [19]. In the clinical setting, radiation damage occurs via induction of apoptosis and inflammation in the target organ; however, in cases of extraneous sources of radiation, this may also occur non-specifically in untargeted tissues. The underlying mechanism of radiation retinopathy is photoreceptor necrosis and mitochondrial destabilization within retinal cells, whereby the damaged mitochondria release ROS which further damage the retina, resulting in a vicious cycle promoting cell death [20]. The effects of intraoral [21] and intramuscular [22] JP4-039 as a radiation mitigator have been described extensively in mice, showing reduced damage from radiation locally in the oral cavity, as well as in blood cell lines after total body irradiation. Plant-derived antioxidants such as crocetin and corticosteroid agents have also been shown to suppress radiation-induced inflammation in retinal diseases [23,24], but to our knowledge, no studies examining the intraocular effects of JP4-039 administered intravitreally have previously been reported.

In our study, we used JP4-039 via a novel route of administration (intravitreal), aiming to determine its local effects in mitigating radiation damage in the retina. We found that intravitreal JP4-039 reduces the acute effects of radiation on the mouse retina by reducing the level of apoptosis as well as decreasing inflammatory cell migration into the retina.

Fluorescent microscopy observations of BODIPY-labeled JP4-039 showed that this molecule can be taken up by retinal cells, most likely influencing intracellular organelles such as mitochondria, which are involved in ROS formation after radiation damage. The nitroxide function of JP4-039 enables it to readily neutralize ROS and scavenge excess electron release from mitochondria, thus likely preventing the destabilization of mitochondrial membranes and maintaining redox homeostasis in the cells [18]. The process by which JP4-039 is absorbed by retinal cells is still unknown, but we speculate that it may occur via endocytosis as well as passive transfer through cell membranes.

Intravitreal JP4-039 injection demonstrated a statistically significant reduction in apoptosis in mouse retinal cells irradiated with a dose of 20 Gy. The ability of JP4-039 to inhibit apoptosis induced by radiation may be due to its chemical properties, since it can cycle between nitroxide, hydroxylamine, and oxoammonium moieties, thus neutralizing ROS and helping to conserve the pool of natural antioxidants such as superoxide dismutase, catalase, and glutathione reductase within the retinal cells [14]. An increase in the pool of endogenous antioxidants would scavenge ROS, thereby stabilizing mitochondrial membranes and reducing the adverse effect of radiation-induced oxidative stress. This effect of JP4-039 has not been previously described, and further study is needed to evaluate the exact processes occurring within the mitochondria themselves.

It is well known that oxidative stress is associated with a recruitment of inflammatory cells [25,26]. Our findings suggest that the intraocular injection of JP4-039 partially inhibits the migration of neutrophils and macrophages into the retina 120 h after irradiation. This may suggest an anti-inflammatory role for JP4-039, perhaps mediated by reduction in oxidative stress, as GS-nitroxides have been shown to exhibit strong anti-inflammatory properties [18,19]. The aim of the 120-h timepoint was to assess the effect of JP4-039 on inflammatory cell migration into the retina after radiation damage, rather than to describe the peak levels of apoptosis, which are currently unknown in animal retinal models [27]. However, Hiroshiba et al. showed that leukocytic pooling in the irradiated rat retina occurred around 7 days after a 20 Gy dose of radiation [28]. Our data show that intravitreal injection, a routine ophthalmic clinical drug delivery route, may allow JP4-039 to specifically target the site of radiation damage, such as retinopathy, potentially allowing for a higher local concentration and reduced risk of systemic side-effects. Should future studies further support JP4-039’s protective role, its translation to clinical practice could be easily applicable for the treatment of radiation retinopathy after safety analysis of the drug in clinical trials.

Potential limitations of our study include the lack of dose-dependent data related to the effects of JP4-039. The lack of other methods to measure apoptosis, such as mitochondrial membrane potential, may have also limited our study’s ability to accurately demonstrate early apoptosis as well as the involvement of the mitochondrial pathway in radiation-induced apoptosis. Additional testing to indicate mitochondrial health following the administration of JP4-039 may be helpful in further demonstrating the beneficial effects of JP4-039 in radiation retinopathy.

In summary, we found that intravitreal injection of JP4-039 significantly reduced apoptosis and inflammatory cell migration in irradiated mouse retinas. More studies are needed to assess the intraocular effects of this electron and ROS scavenger in animal studies prior to examining its effect in the human retina. However, our studies serve as a significant proof-of-concept for further preclinical investigations of this class of ocular therapeutics.

## 4. Materials and Methods

### 4.1. Ethical Statement

This study was approved by the University of Pittsburgh Institutional Animal Care and Use Committee (IACUC; Protocol #21028741). Veterinary care was provided by the University of Pittsburgh Division of Laboratory Animal Resources. Handling of experimental animals was ethical and in compliance with institutional and ARVO (The Association for Research in Vision and Ophthalmology, Inc., Rockville, MD, USA) guidelines. The University of Pittsburgh is approved by the AAALAC (Association for Assessment and Accreditation of Laboratory Animal Care International).

### 4.2. Preparation of JP4-039 for Intravitreal Injections

JP4-039 (4.0 mg) was added to 1 mL of 30% 2-hydroxypropyl-β-cyclodextrin, placed on a hotplate, and stirred at 50 °C until dissolution. A total of 10 µL (0.004 mg/µL) of BODIPY-labeled JP4-039 (treatment) or 2-hydoxypropyl-β-cyclodextrin (vehicle) was used in each of the studied eyes as described below.

### 4.3. Intravitreal Injections and Eye Irradiation

Intravitreal injections were performed after anesthetizing the mice with 2% isoflurane. Observing the mice under a Nikon SMZ645 dissecting microscope (Nikon Metrology, Inc., Brighton, MI, USA), a 32-gauge needle was inserted into the vitreous cavity of the right eye, allowing 10 to 12 µL of vitreous fluid to drain out [29]. The 32-gauge needle was removed, and a new 32-gauge needle was used to inject 10 µL of either the treatment (0.004 mg/µL JP4-039) or vehicle (2-hydoxypropyl-β-cyclodextrin) into the vitreous cavity. The mice were allowed to recover from the isoflurane for thirty minutes before being re-anesthetized using intraperitoneal injections of 70 mg/kg Nembutal. They were then irradiated using a TrueBeam^®^ STx linear accelerator (Varian Medical Systems, Inc., Palo Alto, CA, USA) by placing the mouse on its side with the experimental right eye facing up. Then, a 2 × 3 cm tissue-equivalent bolus that was 1 cm thick was placed over the face covering the right eye, a 6 mm cone was placed on the bolus, and a dose of 20 Gy was applied to the experimental right eye using a 6 MV photon beam. Radiation exposure to deliver 20 Gy was two minutes. Due to the positioning of the mice, the left eyes received unmeasured amounts of radiation; therefore, we used only the right eye in our experiments. After irradiation and prior to further testing, the mice were sacrificed, and the experimental right eyes were enucleated.

### 4.4. Experimental Animals

**Figure 5:**
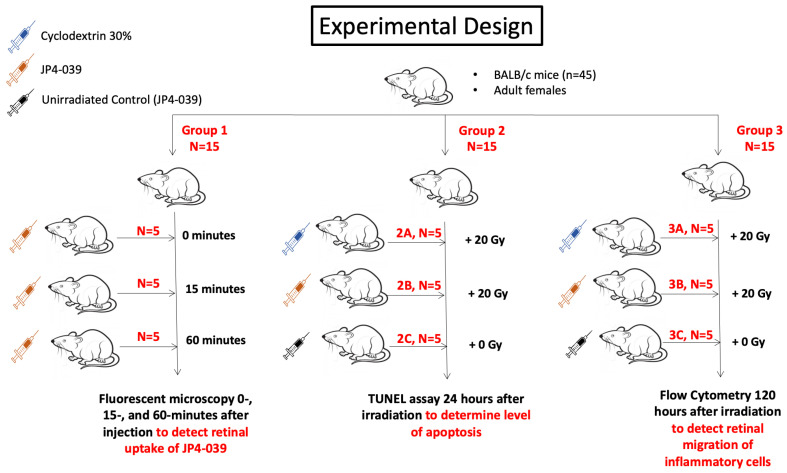
Experimental design. Three groups of BALB/c mice were included. Each group had 15 mice. In Group 1, all mice were injected with JP4-039, and retinal uptake was measured 0, 15, and 60 min after injection. In Group 2, fifteen BALB/c mice were injected intravitreally with 30% 2-hydoxypropyl-β-cyclodextrin alone (2A, *n* = 5 mice) or with JP4-039 (2B, *n* = 5 mice), and were irradiated with 20 Gy. The third subgroup (2C, *n* = 5 mice) was injected with JP4-039 but did not receive radiation. All mice from Group 2 (*n* = 15) were sacrificed and enucleated 24 h later, retinal cell suspensions were made, and apoptosis was measured using a TUNEL assay. In Group 3, fifteen BALB/c mice were injected intravitreally with 30% 2-hydoxypropyl-β-cyclodextrin alone (3A, *n* = 5 mice) or with JP4-039 (3B, *n* = 5 mice), and were then irradiated with 20 Gy to the injected eye. The third subgroup (3C, *n* = 5 mice) was injected with JP4-039 but did not receive radiation. All mice from Group 3 (*n* = 15) were sacrificed and enucleated 120 h later, retinal cell suspensions were made, and flow cytometry was used to detect retinal migration of inflammatory cells.

Forty-five 10- to 12-week-old female BALB/c mice (Taconic Biosciences, Germantown, NY, USA) were used, as shown in Figure 5. One eye per mouse was used. In Group 1, fluorescent microscopy was used to determine retinal uptake of 10 µL (0.004 mg/µL) intravitreally injected BODIPY-labeled JP4-039 (treatment) at 0, 15, and 60 min after injection (*n* = 5 mice per timepoint). In Group 2, a TUNEL assay was performed 24 h after injection of 10 µL of either BODIPY-labeled JP4-039 (0.004 mg/µL) or 2-hydoxypropyl-β-cyclodextrin (vehicle) to investigate the percentage of retinal cells remaining after irradiation with 20 Gy. There were 3 subgroups of 5 mice each: 2A (vehicle with radiation), 2B (treatment with radiation), and 2C (treatment without radiation; control). In Group 3, flow cytometry was performed 120 h after intravitreal injection and irradiation in 3 subgroups of 5 mice each: 3A (vehicle with radiation), 3B (treatment with radiation), and 3C (treatment without radiation; control). The studied eye of each mouse was then enucleated, and the retinas in each subgroup were extracted and converted into a single cell suspension. The cells were stained with antibodies for N1 and N2 neutrophils and M1 and M2 macrophages and analyzed by flow cytometry to determine the number and percentage of cells per group. Inflammatory cell migration was measured at 120 h after irradiation in order to allow time for the eye to mount a response to the cellular damage induced by the radiation [28].

### 4.5. Uptake of JP4-039 in the Retina

To examine the ability of the retina to absorb JP4-039 [30], BODIPY-labeled JP4-039 was injected intravitreally into 15 mice, and was measured at 0, 15, and 60 min after injection (*n* = 5 mice per timepoint) (Group 1). At the designated timepoints, the eyes of the mice were processed for fluorescent microscopy. Under anesthesia, the experimental eye was enucleated and fixed in 4% paraformaldehyde for 30 min at room temperature and was subsequently placed in 30% sucrose for further processing before being embedded in an O.C.T. Compound (Tissue-Tek, Sakura Finetek, CA, USA) and flash-frozen in LN_2_-chilled isopentane. The eyes were dissected into 12 µm sections using a Leica CM 3050 S Cryostat (Leica Biosystems, Nussloch, Germany) and imaged with an Olympus DP80 camera on an Olympus IX83 inverted epi-fluorescent microscope running CellSens software (CellSens Dimension, Version 3.2, Olympus America Inc., Melville, NY, USA). The average fluorescence in the green channel was measured in 12 µm sections spanning from the outer edge of the choroid to the inner retina. Within each image, the pixel intensity of three 500 × 70-pixel regions of the retina were measured using MetaMorph software (v10.1 Molecular Devices, San Jose, CA, USA). The three measurements on each image were averaged to produce a single representative intensity profile for each retina.

### 4.6. Single Cell Suspension of Retina Cells

Processing and staining of retinal tissue for flow cytometry was performed as previously described by Gurley et al., with minor modifications [31]. In brief, the retinas in each subgroup of Groups 2 and 3 were isolated, minced, and placed in liberase enzyme in sterile water for 20 min at 37 °C.

### 4.7. Measurement of Retinal Apoptosis following Irradiation of the Eye

In Group 2, fifteen BALB/c mice were injected intravitreally with 30% 2-hydoxypropyl-β-cyclodextrin alone (2A, *n* = 5 mice) or with JP4-039 (2B, *n* = 5 mice), and were then irradiated with 20 Gy. The third subgroup (2C, *n* = 5 mice) was injected with JP4-039 but did not receive radiation (controls). All mice from Group 2 (*n* = 15) were sacrificed 24 h later. The experimental (right) eye was removed, and the retinas were made into a single cell suspension and pooled as described above. The suspensions were stained for apoptosis using a TUNEL assay (Apo-Direct Apoptosis Detection Kit, Thermo Fisher Scientific, Inc. Waltham, MA, USA) to assess for programmed cell death in the retina. The assay was performed according to package instructions. TUNEL-positive retinal cells were counted using a Becton Dickinson LSR Fortessa I flow cytometer (Becton, Dickinson and Company, Franklin Lakes, NJ, USA) and compared against the total number of cells staining positive for 4,6-diamidino-2-phenylindole (DAPI; dilution,1:1500; Roche^®^ Life Science Products, Sigma-Aldrich, Inc., St. Louis, MO, USA) for determination of the percentage of apoptotic cells.

### 4.8. Migration of N1 and N2 Neutrophils and M1 and M2 Macrophages into the Retina

In Group 3, fifteen BALB/c mice were injected intravitreally with 30% 2-hydoxypropyl-β-cyclodextrin alone (3A, *n* = 5 mice) or with JP4-039 (3B, *n* = 5 mice), and were then irradiated with 20 Gy to the injected eye. The third subgroup (3C, *n* = 5 mice) was injected with JP4-039 but did not receive radiation (controls). All mice from Group 3 (*n* = 15) were sacrificed 120 h later. To detect retinal migration of inflammatory cells, the experimental eye was enucleated, and the retinas from each subgroup were made into a single cell suspension as described above. The digested retinas were incubated with the following antibodies: (1) Alexa Fluor^®^ 700 anti-mouse/human CD11b Antibody, Cat. No.: 101222 (BioLegend, San Diego, CA, USA); (2) PE/Cyanine7 anti-mouse CD45 Antibody, Cat. No.: 103113 (BioLegend, San Diego, CA, USA); (3) APC anti-mouse Ly6C antibody, Cat. No.: 128016 (BioLegend, San Diego, CA, USA); (4) V450 anti-mouse Ly6G antibody, Cat. No.: 560603 BD Horizon (Becton, Dickinson and Company, Franklin Lakes, NJ, USA); and (5) FITC anti-mouse CD206 antibody, Cat. No.: 141703 (BioLegend, San Diego, CA, USA).

The cells were washed in PBS for 10 min, centrifuged at 5000× *g* for 5 min, and resuspended in 500 µL of PBS. The cells were analyzed by flow cytometry using the Becton Dickinson LSR Fortessa I (Becton, Dickinson and Company, Franklin Lakes, NJ, USA) to isolate the monocytes from which the number and percentage of cells positive for N1 and N2 neutrophils, as well as M1 and M2 macrophages, were determined.

### 4.9. Statistics

Statistical analyses were performed using GraphPad Prism 9.3.0.463 (GraphPad Software, Boston, MA, USA). Comparisons between the groups were performed using Analysis of Variance (ANOVA), while those between two groups were performed using Student’s *t*-test.

## Figures and Tables

**Table 1:** Number of N1, N2, M1, and M2 cells found in the retinas of the mice shown in Figure 4. The number of N1, N2, M1, and M2 cells were counted by flow cytometer analysis as described in Figure 4. *: *p* values are shown in the table comparing irradiation-without-treatment (20 Gy + cyclodextrin) and irradiation-with-treatment (20 Gy + JP4-039) mice to treatment-only (0 Gy + JP4-039) mice. #: *p* values comparing irradiation-with-treatment mice (20 Gy + JP4-039) and irradiation-without-treatment mice (20 Gy + cyclodextrin). Results are shown as mean ± standard error of the mean.

Group	N1 Cells	N2 Cells	M1 Cells	M2 Cells
0 Gy + Cyclodextrin	322 ± 156	195 ± 100	1444 ± 92	22 ± 8
20 Gy + Cyclodextrin	72,224 ± 4926 * *p* < 0.0001	13,943 ± 8365	26,617 ± 1056 * *p* < 0.0001	330 ± 264
20 Gy + JP4-039	31,636 ± 12,056 * *p* = 0.0302 # *p* = 0.004	8261 ± 5163	25,442 ± 1194 * *p* = 0.0006 # *p* = 0.0396	99 ± 66

## Data Availability

Data presented in this article is available upon request from the corresponding author.
